# A Systematic Review of Exercise Intervention Program for People With Substance Use Disorder

**DOI:** 10.3389/fpsyt.2022.817927

**Published:** 2022-03-11

**Authors:** Zhilei Zhang, Xiujuan Liu

**Affiliations:** ^1^Department of Physical Education and Health, Heze University, Heze, China; ^2^Department of Politics and Law, Heze University, Heze, China

**Keywords:** drug dependence, exercise intervention, essential factor, mechanism, plasticity

## Abstract

Addiction has been attributed to development of habit-based neural circuits that promote continued substance use despite a conscious wish to abstain. The goal of this study was to determine if physical exercise could serve as an alternative habit to replace habitual substance use, and whether this exercise intervention methods differed for opioid vs. amphetamine Dependents. A total of 14 randomized controlled experimental literatures on exercise intervention in people with opioid and amphetamine use disorder were screened, the 14 literature included 4 opioids and 10 amphetamines. From the 14 literature, the information of intervention program elements were counted, respectively. Independent sample *t*-test was used to compare the similarities and differences between the two intervention methods, and intervention mechanism of dependents were discussed. All rehabilitation exercises for opioid dependents use aerobic exercise, while most rehabilitation exercises for amphetamine dependents use aerobic exercise, and a few use aerobic and anaerobic mixed exercise. There is no significant difference in exercise time, exercise frequency and cycle between the two intervention schemes (*P* > 0.05). The rehabilitation indicators of opioid and amphetamine dependents generally include psychological indicators and physiological indicators, and most of the tests mainly focus on measuring psychological indicators such as mood and drug craving of dependents. The goal of exercise intervention for opioid and amphetamine dependents is similar, the first is to improve mood, reduce craving, improve sleep, and the second is to enhance physical fitness. In the treatment of Substance use disorder, exercise intervention can be used as an auxiliary treatment. Exercise intervention emphasizes low intensity and high frequency. Exercise intervention tends to cultivate long-term exercise habits or exercise lifestyle. Based on this “habit” mechanism, exercise can complete the substitution of material dependence.

## Introduction

Drug addiction is also known as severe substance use disorder (SUD) or substance dependence. The treatment for this kind of substance use disorder costs a lot every year. Based on the psychological and social mechanisms, Weinstock et al., recommend exercise as an adjuvant therapy for opioid treatment (1). Physical activity and special exercise are a potential non-pharmacological therapy for addiction (2). Exercise intervention, which is an inexpensive and direct strategy, may have multiple benefits for mental and physical health.

A cross-sectional survey of middle school students across the United States showed that the decrease in the use of alcohol, cigarettes, and marijuana among students over a period of time was associated with the increase in exercise volume, and it was believed that the participation of exercise and sports teams had a synergistic effect in reducing the use of cigarettes and marijuana (3). A survey of similar high school students found that 80% of the reduction in illegal drug use was related to sports participation, and participation in sports reduced the overall risk of illegal drug use (4). A region at the base of the brain striatum, the nucleus accumbens, is the key zone that mediates the rewarding effects of drugs such as amphetamine and cocaine, which act directly by increasing the levels of dopamine at this site (5). Animal experiments showed that physical activity reduced animals’ self-administration of cocaine, and physical activity might be an effective intervention in substance use disorder prevention planning (6). Animal experiments have shown that running and chronic cocaine have a common induction mechanism in the brain’s reward pathways (7). Individuals addicted to alcohol crave alcoholic beverages, spend time seeking alcohol despite negative consequences and eventually drink to intoxication. With prolonged use, control over alcohol seeking devolves to dopamine-dependent mechanisms implicated in habit learning and individuals in whom alcohol seeking relies more on these mechanisms are more likely to persist in seeking alcohol despite the risk of punishment (8). Clinical studies have shown that exercise can be used as a potential intervention tool for substance use disorder, producing beneficial and durable protection in all stages of substance use disorder. Exercise can be used as an alternative non-drug reward to compete with drugs and reduce the possibility of their use (9). Exercise increases dopamine concentration and activates dopamine receptor, which has the same reward pathway as drug abuse physiologically (10). The duration of positive emotional state produced by exercise exceeds the duration of exercise (11). In view of the key role of dopamine in the process of addiction, the mechanism of exercise’s influence on dopamine signaling in the reward pathway can obviously be used as an adjuvant treatment for dependent behaviors. Exercise can regulate various neurotransmitter systems and intracellular signal transduction, increase the level of brain-derived neurotrophic factors, promote nerve regeneration and glial cell regeneration, regulate genetic apparent changes, etc., enhance the brain’s resistance to addictive substance damage, reduce the brain’s thirst for drugs, and prevent relapse (12). At present, there are many theories about how exercise produces beneficial effects. Although it is not completely clear, it can be determined that physical activity can be used as preventive intervention for drug abuse by improving stress response and emotional regulation (13). Other possible mechanisms include increased socialization, and alternative behaviors that exercise a healthy lifestyle. Long-term studies have shown that for female individuals who rely on amphetamine type stimulus, Taijiquan exercise may change their lifestyle and improve their abstinence ability (14).

Effective substance abuse exercise intervention is a kind of exercise prescription with important clinical significance, which needs theoretical support and continuous accumulation of experience. At present, traditional drugs generally include opium, heroin and other opioids, while new drugs generally include methamphetamine and other amphetamine drugs. The exercise type, time, cycle, frequency and other exercise elements and treatment indicators of the exercise intervention prescriptions of the two types of substance abusers are counted, respectively, sorted and analyzed, and the two types of exercise intervention methods are compared to explore the action mechanism of exercise in substance abuse intervention, which is helpful to understand and formulate a precise exercise treatment scheme for substance abuse.

## Materials and Methods

**FIGURE 1:**
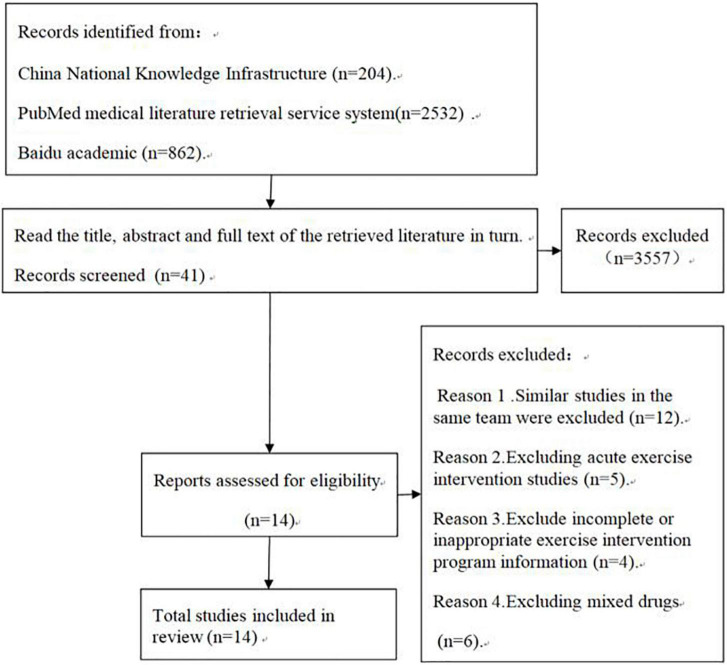
Flow diagram of literature retrieval.

The follow Table 1 for literature sources. The flow diagram of literature retrieval is shown in Figure 1.

**TABLE 1:** Literature retrieval list.

Databases	China National Knowledge Infrastructure retrieval, PubMed medical literature retrieval service system retrieval, Baidu academic retrieval
Time frame (year x-y)	2010–2020
Key words of literature retrieval	illicit drugs, opioids, opium, heroin, cocaine, amphetamine, methamphetamine, drug dependence, drug rehabilitation, drug, clinical trial, randomized controlled trial, exercise, sports, motion, and detoxification
Search content	Title and abstract
Criteria for inclusion and exclusion	The included literature must meet the following conditions at the same time: first, the subjects of the study are opioid or amphetamine drug dependents. Second, the intervention measures are exercise. Third, the description of exercise intervention program is more detailed and comprehensive. Fourth, based on the Consolidated Standards of Reporting Trials statement (CONSORT statement), the 25 items of CONSORT statement were checked The following studies were excluded: First, similar studies in the same team. Second, acute exercise intervention research. Third, incomplete or inappropriate exercise intervention information. Fourth, the intervention experiment of mixed drugs

### Statistical Analyses

The statistical data of various elements of the exercise intervention program for opioid or amphetamine dependents were input into spss20.0 software, the two intervention schemes used independent sample *t*-test to compare the numerical data in exercise time, exercise frequency and cycle. Other descriptive statistics. The addition and subtraction standard deviation of the mean of statistical symbols showed $\bar{\text{x}}$ ± S, and *P* < 0.05 showed significant difference.

## Results

The 14 literature included 4 opioids and 10 amphetamines. From the 14 literature, the information of intervention program elements of opioid and amphetamine drug dependents were counted, respectively, including age (years), exercise time, exercise frequency, cycle, exercise intensity, exercise content, and main rehabilitation indicators. Table 2 (15–18) shows the information list of intervention program elements for opioid dependents. Table 3 (19–28) shows the information list of the intervention program elements of amphetamine-type dependents. Table 4 shows the comparison of the two types of intervention program elements. There is no significant difference in exercise time, exercise frequency and cycle between the two intervention schemes (*P* > 0.05). The two types of subjects are young and middle-aged. The intervention time of exercise is mainly 30–50 min, the frequency of exercise is generally more than 3 times, and the exercise cycle is generally more than 3 months. Most studies have reported the control range of exercise intensity, which is generally medium and low intensity and does not exceed 80% of the maximum heart rate. All rehabilitation exercises for opioid dependents use aerobic exercise, while most rehabilitation exercises for amphetamine dependents use aerobic exercise, and a few use aerobic and anaerobic mixed exercise. The rehabilitation indicators of opioid and amphetamine dependents generally include psychological indicators and physiological indicators, and most of the tests mainly focus on measuring psychological indicators such as mood and drug craving of dependents.

**TABLE 2:** List of information on exercise intervention programs for people with opioid use disorder.

Literature	Average age (years)	Time (minutes)	Frequency (times/week)	Cycle (days)	Intensity	Content	Index
Brown et al. (15)	38.3	30	3	84	69% HRmax	Aerobic exercise	Heart rate, blood pressure, metoprolol, BMI, and body fat
Li et al. (17)	30.29	60	3.5	180	Not reported	Taijiquan	Blood cells, liver function, kidney function, PAS, and HRSD
Smelson et al. (16)	36	15	3	14	Not reported	Qigong	STAI, BDI, and VAS
Zhuang et al. (18)	29.13	50	5	180	Not reported	Yoga	POMS and SF-36

*PAS, heroin withdrawal symptom rating scale; HRSD, Hamilton Depression Scale; STAI, state trait anxiety scale; BDI, Beck Depression Scale; VAS, Visual Analog Craving Scale; POMS, emotional state profile; SF-36, quality of life.*

**TABLE 3:** Information list of exercise intervention programs for people with amphetamine use disorder.

Literature	Average age (years)	Time (minutes)	Frequency (times/week)	Cycle (days)	Intensity	Content	Index
Dolezal et al. (19)	33	60	3	56	Lactate threshold heart rate -HRmax	Jogging and resistance training	HRV_‵_VO2max
Richard et al. (53)	31.7	60	3	56	60–80% HRmax	Aerobic exercise and resistance training	BDI and BAI
Li et al. (21)	35.4	50	7	168	Not reported	Rehabilitation exercises and functional training	CogState
Robertson et al. (22)	29	60	3	56	Not reported	Jogging and resistance training	Utilization ratio of D2/D3 receptors in striatum
Zhu et al. (23)	37.47	50	5	84	100次/分钟	Taijiquan	Body fat, balance, and QOL-DA
Wang et al. (54)	32.20	35	3	84	65–75% HRmax	aerobic exercise	VO2max_‵_HAMA, BDI, and VAS
Zhang et al. (55)	36.49	40	3	84	65–75% HRmax	Bicycle, jogging, and skipping rope	CogState and total antioxidant capacity of serum
Liang et al. (26)	27.1	40	3	90	Moderate intensity	Yoga and aerobics	QOL-DA, SDS, and SAS
Lu et al. (27)	31.47	90	5	84	40–80% HRmax	Moderate intensity exercise and resistance training	SCL-90, SDS, SAS, VAS, and blood index
Liu et al. (28)	18–35	30	2	70	65–75% HRmax	Dance and power bicycle	LFPQ and Clue task

*HRmax, maximum heart rate; HRV, heart rate variability; VO2max, maximum oxygen uptake; BDI, Beck Depression Scale; BAI, baker anxiety scale; CogState, cognitive scale; QOL-DA, quality of life of drug addiction; HAMA, Hamilton Anxiety Scale; VAS, Visual Analog Craving Scale; SDS, self-rating depression scale; SAS, Self-Rating Anxiety Scale; SCL-90, symptom checklist 90; LFPQ, Leeds Food Preference Questionnaire.*

**TABLE 4:** Comparative table of exercise intervention programs for people with opioid and amphetamine use disorder, ($\bar{\text{x}}$ ± S, *n* = 12).

Exercise elements	OUD (*n* = 4)	AUD (*n* = 1)	< *cps*:*it* > *t* < /*cps*:*it* >	*P*
Time (minutes)	37.50 ± 22.17	51.50 ± 17.32	–1.27	0.23
Frequency (times)	3.63 ± 0.95	3.70 ± 1.49	–0.09	0.93
Period (days)	114.50 ± 80.85	83.20 ± 32.72	1.07	0.31
Intensity (reporting rate)	1(25%)	8(80%)		
Sports content (composition ratio)	100% aerobic	100% aerobic		
	0% anaerobic	40% anaerobic		
Intervention indicators (composition ratio)	50% physiological index	40% physiological index		
	75% psychological index	90% psychological index		

*Sports content (composition ratio) and intervention index (composition ratio) are multiple choices, people with opioid use disorder (OUD), and people with amphetamine use disorder (AUD).*

## Discussion

### Exercise Intervention Programs

Amphetamine-type stimulants (ATS) are obtained by modifying the chemical structure of ephedrine, which can damage the nervous and cardiovascular system in many ways (29). The psychological hazards of methamphetamine include amphetamine psychosis, depression, suicide, anxiety, and violence, while the physical hazards include cardiovascular and cerebrovascular diseases, dependence and blood-borne virus transmission (30). Methamphetamine-dependent subjects showed extensive depressive symptoms, with an average score of mild to moderate severity, and adverse mental symptoms were also common (31). It has been generally accepted that physical exercise is beneficial to health, the benefits of physical exercise to the brain have also attracted people’s attention. From the rehabilitation indicators of drug dependents involved in exercise, the first is to improve mood, reduce craving and other auxiliary treatments to improve mental health, quality of life and delay disease development, and the second is to enhance physical fitness and monitor cardiovascular system function.

How can exercise produce the greatest psychological effect? EBgrer et al. (32) put forward an experimental model to promote psychological benefits: aerobic exercise, no competition between people, closed activities, and moderate intensity, at least 20 or 30 min, and regular practice (33). Most of the exercise intervention programs for the dependent people are low-intensity, multi-frequency exercises with flexible exercise time, which meets the above requirements. In order to facilitate practical operation, intervention programs generally use heart rate indicators to control and measure exercise intensity, and exercise risk prevention and control is carried out at no higher than a specific heart rate. Drug-dependent patients have low aerobic capacity, so it is very important for them to receive physiological treatment in clinic. Some studies suggest that high-intensity interval training should be taken as a part of clinical practice (34). However, there is a risk of cardiovascular disease. In practice, aerobic exercise is generally dominant, which may be related to exercise risk control. Studies have shown that there is no significant difference between low-intensity, medium-intensity and high-intensity exercise to intervene substance abuse (nicotine, illegal drugs) (35). There are also studies that moderate-intensity aerobic exercise is the best exercise intensity for methamphetamine (commonly known as meth) dependents to recover (36). Behavioral research shows that the influence of acute exercise on cognitive performance and brain response varies with different exercise intensity: in general, the smaller the exercise intensity, the better the improvement effect (37). Relevant research conclusions are inconsistent, and more evidence is needed. It is worth noting that the physical quality of those who depend on it is generally poor, and the heart and lung functions related to exercise are generally seriously deficient. The safety risks will be very prominent if you take intensive physical exercise.

Exercise intervention is an auxiliary treatment. Illegal Substance use disorder, a serious social problem, urges researchers to innovate methods for rehabilitation. Meta-analysis provides strong evidence, which shows that physical exercise is an effective method for adjunctive treatment of alcoholics, nicotine and illegal drug dependents, and also reduces withdrawal symptoms (35). Exercise may have many different mechanisms, which are beneficial to the rehabilitation of drug-dependent patients. First of all, exercise can help people experience positive emotions, and depression symptoms are associated with poor treatment results. In addition, exercise can alleviate sleep disorders, increase self-esteem and reduce stress response, which may help reduce the risk of relapse of drug-dependent patients, so exercise is used as an intervention measure to prevent relapse of Substance use disorder (15). From more data seem to support the potential benefit of physical exercise as an adjunct in Substance use disorder rehabilitation process now (38). To examine, from behavioral perspectives, the feasibility of applying group-based aerobic exercise as an adjunct to treatment aimed at improving the of substance use disorder patients (39). In addition, Substance use disorder is also a chronic encephalopathy, with complicated etiology, long course of treatment and high relapse rate. Low-intensity and high-frequency exercise intervention may develop long-term exercise habits and exercise lifestyle, which is beneficial for drug dependents to return to society.

Substance use disorder treatment calls for individualized treatment plan, increases the initiative of participating in treatment, and provides more comprehensive care for patients. Investigation shows that during early rehabilitation, the rate of regular exercise is low, and the level of interest in sports is high, so the exercise program can be made according to individual’s unique preferences (40).

### Exercise Intervention “Habit” Mechanism

Repeated medication leads to the individual changing from reward effect to habitual mode (41). Using drug can improve negative emotions such as anxiety, depression and despair, so some people tend to abuse drugs. Most drug abusers have obvious mental disorders and psychological problems, lack trust in others and society, live emptiness, and escape through drug abuse. Although the causes of this phenomenon are very complex, drug abuse is obviously a comprehensive physiological and behavioral response of the dependent (42). Repeated medication forms the reward pathway of “craving-driving-behavior-reward,” and its neural structure is mainly located in the dopamine (DA) system at the midbrain edge, which is the core pathway to form reward effect (43). After the dependents use illegal drugs, the drugs directly stimulate the nervous system, which makes people feel very happy and temporarily get rid of or forget the troubles in reality. Because, they meet the physical and mental needs of the abusers, they form positive reinforcement. However, after the lack of drug stimulation, the dependent people have severe discomfort of withdrawal symptoms, strong psychological craving for drugs, and troubles that can’t escape from reality. This painful experience has formed negative reinforcement. The communication between human spirit and body is very complicated. When such abuse behavior is carried out for a long time, replacing the original brain natural reward or showing new reward, the dependence behavior of euphoria becomes a habit.

**FIGURE 2:**
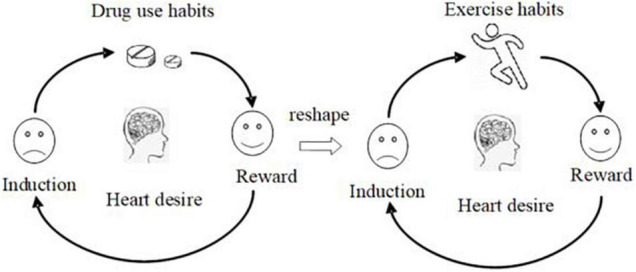
Mechanism diagram of illegal drug use habit replaced by exercise habit.

Every habit has plasticity. Moving from a drug-dependent lifestyle to a drug-free lifestyle, sports services can play an important role. Dependence behavior is an extraordinary hobby and habit (56). By observing, adjusting hints and rewards, we can change our usual behaviors and rebuild our habits. Researchers at the Massachusetts Institute of Technology found a simple neural logic circuit in each habit, which consists of three parts, suggestion, habitual behavior, and reward. The golden rule of changing habits is: using the same hints, offering the same rewards, and inserting new habitual behaviors, you can replace old habits with new habits (44). The formed exercise habit behavior pattern will stay in the brain forever, suppress nerve activity, control habit loop, and suppress bad habits behind the scenes. With the birth of a new pattern, exercise can become a natural activity. In the normal situation, a prepared habit can be held in check, to allow the slower, more reflective, goal-directed process to override it and occur instead (45). Therefore, it is feasible to replace drug abuse habits with exercise habits. Because of the plasticity of midbrain marginal reward pathway, the formation of exercise habits may help to reduce drug abuse dependence and change the reward effect of drug abuse, which may help to reduce the incidence and severity of drug abuse disorder and help to cope with stress successfully (46). Studies have shown that there is a significant positive correlation between the frequency and amount of physical activity and the rehabilitation of Substance use disorder withdrawal (47). There is evidence that the mental health level of female heroin dependents can be improved through the regulation of “exercise habits” (58). Regular exercise to intervene substance dependent people is actually the cultivation of a healthy “exercise habits” (48, 49). Teenagers’ preference for sports may be easier to form habits than adults. Therefore, if people participate in sports and achieve good emotional effects, avoid negative emotions, and play a role in self-defense and self-protection, sports can contribute as an alternative means of drug abuse (57). The “habit” mechanism of exercise intervention in substance use disorder is shown in Figure 2.

Rebuilding habits cannot be done once and for all. People who have successfully quit drinking will relapse when faced with a stressful event again. No matter how many new habitual behaviors they have developed, their habits will never disappear. Its old habits have not disappeared, it has only been replaced by new habits. That is to say, new habits are not created out of thin air, but new habitual behaviors are substituted for old behaviors by splitting habit loops. Behaviorists and psychological scientists advocate treating the abuse of illegal drugs as a bad behavior, that is, paying attention to the important role of psychological rehabilitation of dependents in the process of withdrawal, and striving to strengthen psychological counseling and behavior shaping of dependents (50). New drugs are also called party drugs. In China, most of the behaviors of taking new drugs are group behaviors (51). Collective exercise in sports can establish contact with peers, form a mutually restrained group, and reduce the tendency to use drugs due to self-isolation (52), which is often very meaningful for those who depend on it to quit illegal drugs. In particular, sports can screen and optimize communication groups and reduce the possibility of contact with illegal drugs.

## Conclusion

There is no significant difference in the elements of exercise time, frequency and cycle between opioid and amphetamine dependents (*P* > 0.05). The goal of exercise intervention is similar, the first is to improve mood, reduce craving, improve sleep, and the second is to enhance physical fitness. In the treatment of Substance use disorder, exercise intervention can be used as an auxiliary treatment. Exercise intervention emphasizes low intensity, high frequency, and long-term exercise habit or sports lifestyle. Based on the “exercise habit” mechanism, exercise may complete the substitution of Substance use disorder.

## Limitations

This study is helpful to understand and formulate a precise exercise intervention program for drug abuse, but an important limitation is that the literature review does not evaluate the therapeutic effect of exercise intervention program and does not know which indicators are of therapeutic significance. It is a good solution to evaluate the efficacy of exercise intervention program on individual indicators through meta-analysis.

## Data Availability Statement

The original contributions presented in the study are included in the article/supplementary material, further inquiries can be directed to the corresponding author.

## Author Contributions

ZZ contributed to the idea for the article, organized the study, and wrote the manuscript. XL helped with the analysis. Both authors contributed to the article and approved the submitted version.

## Conflict of Interest

The authors declare that the research was conducted in the absence of any commercial or financial relationships that could be construed as a potential conflict of interest.

## Publisher’s Note

All claims expressed in this article are solely those of the authors and do not necessarily represent those of their affiliated organizations, or those of the publisher, the editors and the reviewers. Any product that may be evaluated in this article, or claim that may be made by its manufacturer, is not guaranteed or endorsed by the publisher.
